# Monoclonal antibody-based localization of major diagnostic antigens in metacestode tissue, excretory/secretory products, and extracellular vesicles of *Echinococcus* species

**DOI:** 10.3389/fcimb.2023.1162530

**Published:** 2023-03-16

**Authors:** Philipp A. Kronenberg, Michael Reinehr, Ramon Marc Eichenberger, Sina Hasler, Teivi Laurimäe, Achim Weber, Ansgar Deibel, Beat Müllhaupt, Bruno Gottstein, Norbert Müller, Andrew Hemphill, Peter Deplazes

**Affiliations:** ^1^ Institute of Parasitology, Vetsuisse and Medical Faculty, University of Zurich, Zurich, Switzerland; ^2^ Graduate School for Cellular and Biomedical Sciences, University of Bern, Bern, Switzerland; ^3^ Department of Pathology and Molecular Pathology, University Hospital Zurich, University of Zurich, Zurich, Switzerland; ^4^ Microbiology and Molecular Biology, Institute of Chemistry and Biotechnology, Zurich University of Applied Sciences, Zurich University of Applied Sciences’ (ZHAW), Wädenswil, Switzerland; ^5^ Department of Gastroenterology and Hepatology and Swiss HPB and Transplant Center, University Hospital Zurich, University of Zurich, Zurich, Switzerland; ^6^ Institute for Infectious Diseases, Medical Faculty, University of Bern, Bern, Switzerland; ^7^ Institute of Parasitology, Vetsuisse Faculty, University of Bern, Bern, Switzerland

**Keywords:** Echinococcus multilocularis, Echinococcus granulosus sensu lato, II/3-10, Em18, Em2, EmG3, antigen B, 2B2

## Abstract

Alveolar (AE) and cystic echinococcosis (CE) are severe parasitic zoonoses caused by the larval stages of *Echinococcus multilocularis* and *E. granulosus sensu lato*, respectively. A panel of 7 monoclonal antibodies (mAbs) was selected against major diagnostic epitopes of both species. The binding capacity of the mAbs to *Echinococcus* spp. excretory/secretory products (ESP) was analyzed by sandwich-ELISA, where mAb Em2G11 and mAb EmG3 detected *in vitro* extravesicular ESP of both *E. multilocularis* and *E. granulosus s.s.* These findings were subsequently confirmed by the detection of circulating ESP in a subset of serum samples from infected hosts including humans. Extracellular vesicles (EVs) were purified, and the binding to mAbs was analyzed by sandwich-ELISA. Transmission electron microscopy (TEM) was used to confirm the binding of mAb EmG3 to EVs from intravesicular fluid of *Echinococcus* spp. vesicles. The specificity of the mAbs in ELISA corresponded to the immunohistochemical staining (IHC-S) patterns performed on human AE and CE liver sections. Antigenic small particles designated as ‘‘spems’’ for *E. multilocularis* and ‘‘spegs’’ for *E. granulosus s.l.* were stained by the mAb EmG3_IgM_, mAb EmG3_IgG1_, mAb AgB, and mAb 2B2, while mAb Em2G11 reacted with spems and mAb Eg2 with spegs only. The laminated layer (LL) of both species was strongly visualized by using mAb EmG3_IgM_, mAb EmG3_IgG1_, mAb AgB, and mAb 2B2. The LL was specifically stained by mAb Em2G11 in *E. multilocularis* and by mAb Eg2 in *E. granulosus s.l.* In the germinal layer (GL), including the protoscoleces, a wide staining pattern with all structures of both species was observed with mAb EmG3_IgG1_, mAb EmG3_IgM_, mAb AgB, mAb 2B2, and mAb Em18. In the GL and protoscoleces, the mAb Eg2 displayed a strong *E. granulosus s.l.* specific binding, while mAb Em2G11 exhibited a weak granular *E. multilocularis* specific reaction. The most notable staining pattern in IHC-S was found with mAb Em18, which solely bound to the GL and protoscoleces of *Echinococcus* species and potentially to primary cells. To conclude, mAbs represent valuable tools for the visualization of major antigens in the most important *Echinococcus* species, as well as providing insights into parasite-host interactions and pathogenesis.

## Introduction

Cystic echinococcosis (CE) and alveolar echinococcosis (AE) are among the most severe parasitic zoonotic diseases. CE is caused by the larval stage of the dog tapeworm, *Echinococcus granulosus sensu lato* (*s.l.*), leading to cysts in primarily the liver and lungs of infected patients (Kern et al., 2017). AE is caused by the larval stage of the fox tapeworm, *Echinococcus multilocularis*. In human AE, the infiltrative and cancer-like metacestode growth is usually fatal if not treated appropriately (Eckert and Deplazes, 2004). AE and CE are responsible for extensive human morbidity with an estimated global 18’400 AE and 188’000 CE cases per year (Torgerson et al., 2015). Human infections caused by the species *E. vogeli* and *E. oligarthra* are rare and the diseases caused by the two species, which are confined to South America, are known as neotropical (polycystic) echinococcoses (Kern et al., 2017). To date, the diagnosis of echinococcosis is based on a combined approach of imaging technologies, serology, molecular methods, and histopathology (Brunetti et al., 2010). Nevertheless, a confirmed early diagnosis of AE and CE remains challenging. The diagnosis is usually based on ultrasound and serology, but the specificity of these methods is limited. Given that fine needle biopsies are relatively invasive and only molecular or histopathological examination can confirm the disease, a genuine disease confirmation is difficult (Brunetti et al., 2010; Reinehr et al., 2020). Nevertheless, in clinical settings, the diagnostic approach by using radiology combined with serology is often sufficient to have enough evidence of echinococcosis. These insights are evident as demonstrated by experiences of the largest European center for echinococcosis in Ulm, Germany. In this study, a confirmed diagnosis of AE in a long-term cohort was achieved in only 55% of 312 patients by histopathology or molecular methods. The remaining 45% of patients were classified as probable (36%) or possible (9%) diagnoses, connected to positive radiological findings and/or serology (Grüner et al., 2017).

The metacestodes of *Echinococcus* species are fluid filled vesicles, that are surrounded by a highly glycosylated and acellular wall, the laminated layer (LL). The inside of the LL is lined with a thin germinal layer (GL), which is composed of parasite cells (Thompson, 2017). Metacestodes can proliferate asexually by forming daughter vesicles or cysts, and brood capsules containing protoscoleces. Especially in *E. multilocularis*, this process is locally invasive *in vivo*. Moreover, metacestodes of *E. multilocularis* can be maintained *in vitro*, where they can proliferate into new vesicles either directly from the GL, or by budding of daughter metacestodes from older vesicles (Hemphill et al., 2010). The germinal cells (including primary cells) of the GL are involved in the growth and regeneration of the metacestodes of *E. multilocularis in vitro* (Spiliotis et al., 2008) and can cause metastasis formation *in vivo* (Eckert et al., 1983).

For species-specific immunohistochemical stainings (IHC-S), several monoclonal antibodies (mAbs) were developed. A differential diagnosis of AE and CE in human tissue specimens can be achieved by IHC-S with a dual staining approach with mAbs (Barth et al., 2012; Reinehr et al., 2020). While mAb Em2G11 is specific for *E. multilocularis*, mAb EmG3 recognizes epitopes of *E. multilocularis, E. granulosus s.l.* and *E. vogeli* (Reinehr et al., 2020). Both mAbs share the ability to react with affinity purified Em2 antigen, which is considered to be specific for *E. multilocularis*. The Em2 antigen is one of the major diagnostic antigens used in serology for a specific diagnosis of AE (Gottstein et al., 1983). Both mAb Em2G11 and mAb EmG3 are directed against the metacestode laminated layer and share the ability to stain ‘‘small particles of *E. multilocularis* and/or *E. granulosus*’’ designated as ‘‘spems’’ and ‘‘spegs’’. Spems and Spegs are antigenic remnants, that are found outside of the laminated layer in the host tissue surrounding the lesions (Reinehr et al., 2020; Ricken et al., 2017) and in the bordering lymph nodes (Grimm et al., 2020). The use of mAbs for the diagnosis and characterization of *E. granulosus s.l.* is so far scarce, describing only mAbs against epitopes of Antigen B or Antigen 5 in the cyst fluid. Those mAbs were evaluated for the detection of circulating parasite antigens in the blood but showed low sensitivities in serology (Liu et al., 1993; Siles-Lucas et al., 2017a). To the best knowledge of the authors, a mAb for a species-specific diagnosis of *E. granulosus s.l.* was documented for the first time in this publication (mAb Eg2).

Several antigens and sub-fractions of the metacestode of *E. granulosus s.l.* are well described. One of these antigens is Antigen B (AgB), a major lipoprotein of the cyst fluid with a high genetic homology between all *Echinococcus* species. AgB is an immunogenic lipoprotein with a size of approximately 160 kDa, which under reducing conditions dissociates into subunits of 8, 16 and 24 kDa, suggesting multimers of 8 kDa. These subunits have a wide application in immuno-blots of AgB or cyst/vesicle fluid for the serological diagnosis of AE (Müller et al., 2007) and CE (Poretti et al., 1999; Siles-Lucas et al., 2017a). Furthermore, several recombinant antigens derived from the AgB gene family were developed for serological applications. Therefrom, a tandem repeat of the antigen AgB2, named Ag2B2, seems to have the most potential for the diagnosis of CE patients (Hernández-González et al., 2012). However, like AgB, this antigen does not seem to be species-specific for *E. granulosus s.l.*, with 33% sero-positivity in 60 tested AE patients (Kronenberg et al., 2022). For a specific diagnosis of *E. multilocularis*, antigen II/3 (Synonym Em10) was developed (Vogel et al., 1988). The diagnostic epitopes thereof are Em18 and II/3-10, which are sub-fragments of the full-length antigen II/3 (Felleisen and Gottstein, 1993; Sako et al., 2002). These two antigens are immunologically most likely identical and can be used for a serological parasite-viability assessment after long-term therapy with albendazole in AE patients. A negative II/3-10 or Em18 antibody serology is correlated to a positive treatment outcome and clinical remission in combination with PET-CT (Ammann et al., 2015; Deibel et al., 2022; Gottstein et al., 2019; Hotz et al., 2022). In immunohistochemistry with polyclonal rabbit antibodies directed against the II/3 antigen, the germinal layer and protoscoleces can be visualized. Unfortunately, these stainings have rather high background reactions and are unsuitable for diagnostic purposes or for histopathological viability assessments (Frosch et al., 1991; Felleisen and Gottstein, 1993). Therefore, a mAb reactive with the recombinant and the native epitope of the Em18 and II/3-10 antigen was developed for this study (mAb Em18).

Moreover, several other antigen candidates have been described in the literature with a potential for circulating in the blood system. The most promising antigens are Antigen B and Em2 antigen (Deplazes and Gottstein, 1991; Siles-Lucas et al., 2017a). Up to date, antigen detecting assays are limited in AE and CE diagnostics and are lacking in sensitivity. Most of these assays are based on rather unspecific polyclonal antibodies and have never reached commercial application status (Craig and Nelson, 1984; Gottstein, 1984; Siles-Lucas et al., 2017a). Nevertheless, the mAb Em2G11 can be used for the detection of circulating Em2 antigen in heavily infected accidental intermediate hosts such as dogs or monkeys (Staebler et al., 2006). In one of the earlier studies, sandwich-ELISA detected the Em2 antigen in the supernatant of *in vitro* cultured vesicles, in the vesicle fluid, and in vesicle crude antigen (Hemphill and Gottstein, 1995).

In recent years, substantial research was focused on the description of extracellular vesicles (EVs) in multiple helminth species. Helminths release EVs into their environment, which have various effects that are relevant in parasite-host interactions (White et al., 2023). So far, this work was mainly focused on the characterization of EV-molecules and their specific interactions with host cells and tissues. A helminth-specific EV marker/antibody to study biological functions is so far missing and an EV-protein detecting assay could improve the diagnosis of helminth infections by detecting stable circulating parasite particles (Sotillo et al., 2020). The diagnostic potential of helminth EVs was so far focused on EV-containing RNAs as biomarkers, with limited diagnostic value (Mu et al., 2021). For both *E. granulosus s.l.* and *E. multilocularis*, EVs are described as parts of the cyst or vesicle fluid and are mostly composed of antigens already identified in the metacestode (Siles-Lucas et al., 2017b; Zheng et al., 2017; Zhou et al., 2019). Furthermore, EVs in *E. multilocularis* contain sRNA and are secreted more towards the inner vesicle fluid of metacestodes and are not released to the outside *in vitro*, indicating different populations of *E. multilocularis* EVs (Ancarola et al., 2020). In the present study, we show the binding of two monoclonal antibodies used for diagnostic purposes (mAb EmG3_IgM_ and mAb EmG3_IgG1_) to intravesicular EVs of all *Echinococcus* species evaluated in this study.

The goal of this research is the development and evaluation of new mAbs for an improved characterization and localization of major antigens of both *E. multilocularis*, *E. granulosus s.l.*, and possibly other *Echinococcus* species like *E. vogeli*. Therefore, we have evaluated all available mAbs for their utility in specific immunohistochemical staining and diagnostic applications.

## Materials and methods

### Parasite antigens for the production of monoclonal antibodies

An overview of the terminology and origin of all mAbs is summarized in **Table 1**. The recombinant Em18 antigen (Ito et al., 2002) was provided by Bruno Gottstein from the Institute for Infectious Diseases in Bern, Switzerland, and was used for the generation of mAb Em18. Recombinant 2B2 antigen (Hernández-González et al., 2012) was supplied by Mar Siles-Lucas (Instituto de Recursos Naturales y Agrobiologia in Salamanca, Spain) and was used for the generation of mAb 2B2. To generate mAb AgB, pure vesicle fluid (EmVF) of *in vitro* cultivated *E. multilocularis* vesicles (6-month culture) was aspirated with an insulin syringe (6mm x 31G, BD Veo) after five washing steps in phosphate buffered saline (PBS). Subsequently, the aspirated EmVF was centrifuged (16’000 g, 15 min, 4°C) and the supernatant was used directly for the immunization of mice. The *in vitro* cultivation of *E. multilocularis* vesicles was performed as described elsewhere (Laurimäe et al., 2020). For the generation of mAb Eg2, *in vitro* vesicles of *E. granulosus s.s.* (genotype G1) were cultured as described for *E. multilocularis* and a vesicle crude antigen (EgVC) was used for the immunization (Kronenberg et al., 2022). Both mAb Em2G11 and mAb EmG3_IgM_ were described in other publications (Deplazes and Gottstein, 1991; Reinehr et al., 2020). A further mAb EmG3_IgG1_ against the EmG3 antigen was sourced from an immunization with an *E. multilocularis* vesicle crude antigen (Kronenberg et al., 2022). As a negative IgG1 control antibody for *Echinococcus* spp. epitopes, mAb D.i 36/1 was included, directed towards *Dirofilaria immitis* and *D. repens* adult somatic and ESP antigens (Joekel et al., 2017). As a negative IgM control, mAb A.v 2/1/3 was used, directed against the parasitic nematode *Angiostrongylus vasorum* (Schnyder et al., 2011).

**Table 1:** Terminology and origin of monoclonal antibodies (mAbs).

mAb Terminology	Isotype	Abbrevation	Antigen origin	Parasite	Reference
mAb Em2G11 ‘‘clone G11’’	IgG1	mAb Em2G11	metacestode crude antigen	*E. multilocularis*	Deplazes and Gottstein (1991)
mAb EmG3 ‘‘clone G3’’	IgM	mAb EmG3_IgM_	metacestode crude antigen	*E. multilocularis*	Reinehr et al. (2020)
mAb EmVC ‘‘clone 18/1/1’’	IgG1	mAb EmG3_IgG1_	*in vitro* vesicle crude antigen	*E. multilocularis*	new
mAb Em18 ‘‘clone 34/1/1’’	IgG1	mAb Em18	recombinant Em18 antigen	*E. multilocularis*	new
mAb EmAgB ‘‘clone P3A1’’	IgG1	mAb AgB	*in vitro* pure vesicle fluid	*E. multilocularis*	new
mAb Eg2B2 ‘‘clone 22/1/1’’	IgG1	mAb 2B2	recombinant 2B2 antigen	*E. granulosus s.s.*	new
mAb Eg2 ‘‘clone 3/1/2’’	IgM	mAb Eg2	*in vitro* vesicle crude antigen	*E. granulosus s.s.*	new
mAb D.i ‘‘clone 36/1’’	IgG1	mAb D.i 36/1	somatic and ESP antigen	*Dirofilaria immitis*	Joekel et al. (2017)
mAb A.v ‘‘clone 2/1/3’’	IgM	mAb A.v 2/1/3	ESP antigen	*Angiostrongylus vasorum*	Schnyder et al. (2011)

### Characterization of the selected monoclonal antibodies by ELISA and western blot

Antibody isotypes were determined by using a mouse monoclonal antibody isotyping set (ISO2-1KT, Sigma Aldrich). The evaluation of the binding targets and the cross-reactivity of the mAb panel against parasite antigens is summarized in **Table 2**, whereas a complete description is found in **Supplementary Table 1**. Species- and stage specific binding and potential cross-reactivity of the mAbs was evaluated by using a panel of 54 parasite antigens in direct ELISA. All antigens were derived from the in-house collection of the Institute of Parasitology in Zurich, Switzerland, and coated at 5 μg/ml. Direct ELISA was performed in principle as described by Kronenberg et al. (2022). In this study, a goat anti-mouse-IgG (whole molecule) conjugate (alkaline phosphatase) was used as a secondary antibody at a dilution of 1:5000 (A3562, Sigma Aldrich). For the evaluation of the antigenicity of the applied antigens, serum from a BALB/c mouse infected with 500 viable eggs of *E. multilocularis* with progressive liver AE was used in a dilution of 1:200. Conjugate-controls were applied in every ELISA (secondary antibody only). All mAbs were furthermore tested on two western blots derived from *E. multilocularis* vesicle fluid (Müller et al., 2007) and *E. granulosus s.l.* cyst fluid (Poretti et al., 1999) (**Supplementary Table 1**).

**Table 2:** Antigen binding of monoclonal antibodies on various parasitic antigens in direct ELISA.

Species		mAb	mAb	mAb	mAb	mAb	mAb
G = genotype	number and stage of antigens (Ag)	Em2G11	EmG3^#^	Em18	AgB	2B2	Eg2
*E. multilocularis*	4x metacestode crude Ag	++++	++++	+	++++	+	–
*E. multilocularis*	2x *in vitro* vesicle somatic Ag*	+++	++++	++	+++	+	–
*E. multilocularis*	2x *in vitro* pure vesicle fluid Ag*	–	+	–	++++	++	–
*E. multilocularis*	*in vitro* excretory/secretory (ESP) Ag	++	+++	–	–	–	–
*E. multilocularis*	2x protoscolex crude Ag*	–	+++	++	–	–	–
*E. multilocularis*	affinity purified Em2G11 Ag	++++	+++	–	–	–	–
*E. multilocularis*	2x adult worm crude and ESP Ag	–	–	+	–	–	–
*E. multilocularis*	recombinant Em18 and EmII/3-10 Ag	–	–	++++	–	–	–
*E. granulosus*, G1/G7	2x metacestode crude Ag	–	++++	+	+++	+	++
*E. granulosus*, G1-3	*in vitro* vesicle somatic Ag	–	++++	+	+	–	+++
*E. granulosus*, G1-3	*in vitro* pure vesicle fluid Ag	–	+++	–	+++	+	–
*E. granulosus*, G1-3	*in vitro* excretory/secretory (ESP) Ag	–	++	–	–	–	–
*E. granulosus*, G1-3	protoscolex crude Ag	–	+++	++	+	–	+
*E. granulosus*, G1-G7	5x cyst fluid Ag	–	++++	–	++++	+++	–
*E. granulosus*, G1-3	Antigen B**	–	+++	–	+++	++++	–
*E. granulosus*, G1-3	adult worm crude Ag	–	–	+	–	–	–
*E. granulosus*	recombinant 2B2 Ag	–	–	–	–	++++	–
*E. vogeli*	metacestode crude Ag	–	+++	+	+	+	–
Taenia spp***	5x metacestode crude Ag	–	–	–	–	–	–
Helminths****	12x larval or adult Ag	–	–	–	–	–	–

ELISA OD: - (0.0-0.05)/+ (0.05-0.25)/++ (0.25-0.5)/+++ (0.5-1.0)/++++ (>1.0)/direct ELISA: Coating with antigens, detection of mAbs with an anti-mouse conjugate as described in Material & Methods./^#^Both mAb EmG3_IgM_ and mAb EmG3_IgG1_ had identical results/* European haplotype, E4 (Nakao et al., 2009) and Kyrgyz haplotype, A2 (Alvarez Rojas et al., 2020)/** Antigen B purified from cyst fluid (Poretti et al., 1999)/***crude metacestode antigens of *T. solium, T. hydatigena, T. saginata, T. multiceps*, and *T. crassiceps*/****crude adult or larval antigens of *Fasciola hepatica, Dicrocoelium dendriticum, Shistosoma mansoni, Litomosoides carinii, Onchocerca jakutensis, Trichinella spiralis, Strongyloides ratti, Toxocara canis, Dirofilaria immitis, Dirofilaria repens, Ascaris lumbricoides*, and *Angiostrongylus vasorum*.

### Immunization of mice and generation of hybridoma cell lines

For each immunization and antigen candidate, two NMRI mice were injected subcutaneously in the neck region at day 0 with either 100 μg (native antigens) or 20 μg (recombinant antigens), mixed 1:2 in Freud’s complete adjuvant. Two further booster injections were applied 1:2 in Freud’s incomplete adjuvant at day 21 and 42. Ten days after the second boost, the mice were injected with either 100 μg or 20 μg of antigens (mixed with sterile phosphate buffered saline, PBS) intraperitoneally for three consecutive days. Fusions of mice spleen cells and Ag8 mouse myeloma cells were carried out 24 hours after the last intraperitoneal immunization and subsequent CO_2_ euthanasia of the mice. Murine monoclonal antibodies (mAbs) were produced in principle as described by Eichenberger et al. (2017). It is of note, that only *Echinococcus* genus or species-specific mAbs were further evaluated. In brief, antibody secreting cell lines were screened by ELISA after two weeks in HAT enriched medium (H0262, Sigma-Aldrich) by using the immunized antigens and native antigens of *E. multilocularis* and *E. granulosus s.l*. Positive cell lines that secreted antibodies against the immunized antigens and native antigens in ELISA were submitted to two additional steps of sub-cloning (1 cell per well in HT enriched medium, H0137 Sigma-Aldrich) until stable and antibody secreting cell lines were established. The sub-clones were further proliferated at 37°C and 5% CO_2_ in Iscoves modified Dulbecco medium (I3390), with 5% ultra-low IgG fetal calf serum (F1283), 1% L-glutamine 200 mM (G7513), 0,6% L- Alanyl-L-Glutamine 200 mM (G8541), 0,06% Gentamicin 50 mg/ml (G1397, all Sigma Aldrich), 10% conditioned medium (Ag8 cell medium supernatant) and 2% IL-6 enriched supernatant from X63JI6 cells (gifted from Prof. Antonius G. Rolink, Biozentrum, University of Basel, Switzerland). Antibody-secreting hybridoma clones were further propagated by using CELLine™ Bioreactor Flasks (Z688029, Sigma Aldrich). Antibodies were afterwards purified by HiTrap™ Protein G. or Protein L. columns (GE29-0485-81, GE29-0486-65, Sigma Aldrich) with a Fast Protein Liquid Chromatography (ÄKTA pure) system. Protein G. was used for the purification of murine IgG1 subclasses and Protein L. for IgM subclasses. Murine IgG1 subclasses could not be purified *via* Protein L. columns, despite the binding capacities indicated in the manufacturer’s protocols.

### Immunohistochemical stainings on human AE and CE tissue samples

Liver tissue sections of one AE and one CE patient were identified from the archives of the Department of Pathology and Molecular Pathology at the University Hospital Zurich, Switzerland. Sections were chosen from active parasite tissue, containing laminated layer, germinal layer, brood capsules and protoscoleces of both *E. multilocularis* and *E. granulosus s.l.* The immunohistochemical stainings (IHC-S) were performed in principle as described by Reinehr et al. (2020). The mAbs were diluted according to previous titration studies, which was adapted from ELISA and optimized for IHC-S.

### Isolation of *in vitro* excretory-secretory products and purification of extracellular vesicles

Metacestodes of both *E. multilocularis* and *E. granulosus s.s.* were cultured *in vitro* as previously described (Laurimäe et al., 2020). After 6 months of *in vitro* co-cultivation with 3T3 feeder cells, the vesicles were transferred to fetal bovine serum (FBS) free medium without 3T3-feeder cells. After one week of subsequent culture, the supernatant was collected and concentrated *via* centrifugal filter units at 3000 g for 5 to 15 minutes (10 kDa for extravesicular ESP and 100 kDa for EVs, Merk Millipore, Ultra 15, 910008). In the meantime, the vesicles were washed 5x with PBS and the intravesicular fluid (EmVF and EgVF) was aspirated aseptically as described earlier. Both extravesicular ESP fractions (EmESP and EgESP) and pure intravesicular fluid (EmVF and EgVF) were used for the purification of EVs. As a negative control, concentrated culture medium from 3T3-feeder cells containing 10% FBS was included (CoESP). In addition, *E. vogeli* cyst fluid (EvCF) collected from infected gerbils was used as a proof-of-principle for *Echinococcus* spp. specific epitopes. The EV fractions of the mentioned extravesicular ESP and intravesicular fluid fractions were isolated and purified with a protocol from Eichenberger et al (Eichenberger, 2021), according to MISEV guidelines (Théry et al., 2018; White et al., 2023). In brief, a combination of differential ultracentrifugation followed by Opti-prep^®^ discontinuous gradient (ODG) was used to purify EV fractions (fractions 3 to 8). As a starting material, 500 µl of EgVF, EmVF, and EvCF was used to purify EVs from *in vitro* derived vesicle fluid or cyst fluid. Furthermore, 500 µl of concentrated extravesicular EmESP, EgESP, and CoESP were used for ultracentrifugation. Soluble antigens were retrieved from the supernatant after the first ultracentrifugation step at 110’000 g for 3 hours in PBS. The EV pellet underwent another ultracentrifugation in an Opti-prep^®^ gradient at 110’000 g for 18 hours. Subsequently, the EV containing fractions were carefully collected and the protein content was measured by BCA (23227, Thermo Fisher). Furthermore, the density of the fractions was measured by ELISA. In addition, the particle concentration (particles/ml) and sizes (nm) were measured by tunable resistive pulse sensing on qNano (Izon Science) and by nanoparticle tracking analysis on ZetaView (ParticleMetrix) (Borup et al., 2022). For further approaches, Optiprep^®^ density gradient (ODG) fractions were pooled with a density in an optimal range for helminth EVs (1.09-1.12 g/mL), which corresponds also to the highest protein concentrations. The pooled ODG fractions were used for the detection of EVs in sandwich-ELISA with mAbs, which was also performed with soluble extravesicular ESP from the identical batches. The measurements of particle sizes, EV- and protein concentrations of all fractions are displayed in **Supplementary Table 2**. For *E. multilocularis*, the EVs had an average size of 95.5 nm in vesicle fluid and 115.2 nm in extravesicular ESP. For *E. granulosus s.s.*, the EVs had an average size of 107.3 nm in vesicle fluid and 107.6 nm in extravesicular ESP.

### Antigen binding of mAbs with *in vitro* excretory/secretory products, serum samples, and extracellular vesicles

The extravesicular ESP fractions as described above were collected from *in vitro* cultivated vesicles of two different isolates of *E. multilocularis* (E4 European- and A2 Asian haplotype) (Kronenberg et al., 2022). For *E. granulosus s.s. in vitro* vesicles, two different isolates of the G1 genotype were isolated from infected sheep cysts in Italy (Kronenberg et al., 2022). Serum samples of humans, other primates, dogs and sheep with naturally acquired liver AE or CE, and experimentally infected rodents with secondary intraperitoneal AE or CE were sourced from the anonymized serum collection of the Diagnostic Centre at the Institute of Parasitology, University of Zurich, Switzerland. The antigen detecting sandwich-ELISA was performed similarly as described for the antibody detection in echinococcosis patients by Kronenberg et al. (2022). In brief, purified mAbs were coated at 5 µg/ml. After incubation over-night, blocking and three washing steps, serum samples were diluted 1:2 in blocking buffer solution and *in vitro* derived extravesicular ESP were added at 10 µg/ml in blocking buffer (PBS, pH 7.2, containing 0.02% NaN_3_, 0.05% bovine haemoglobin and 0.3% Tween-20). After one hour in a wet chamber at 37°C and three washing steps, polyclonal rabbit serum directed against *E. multilocularis* and *E. granulosus s.s. in vitro* vesicle crude antigen was diluted 1:500 in blocking buffer. Thereafter, a goat-anti rabbit IgG-AP conjugate (A3937, Sigma Aldrich) was used for the detection of rabbit polyclonal antibodies. All incubation steps were performed for 1 hour at 37°C in a wet chamber and finished with 3 washing steps. The further ELISA procedures (substrate and reading) were performed as described by Kronenberg et al. (Kronenberg et al., 2022). The detection of purified EV fractions was performed as described for extravesicular ESP at a dilution of 10 µg/ml in blocking buffer.

### Immunogold-transmission electron microscopy of LR-White embedded *E. multilocularis* metacestodes

*In vitro* cultured *E. multilocularis* metacestodes were harvested after 6 months, washed in 100 mM sodium phosphate buffer (pH 7.2) and fixed in phosphate buffer containing 3% paraformaldehyde and 0.05% glutaraldehyde for 2 hours at room temperature. They were then washed in PBS, and sequentially dehydrated in ethanol (30, 50, 70, 90 and 100%) and embedded in LR White acrylic resin (Sigma Aldrich) at -20°C, with three changes of resin during 3 days. Polymerization was done at 60°C overnight. Ultrathin sections were cut by using an ultramicrotome (Reichert and Jung, Vienna, Austria) and were placed on 300 mesh formvar-carbon-coated nickel grids (Plano GmbH, Marburg, Germany). After incubating sections on a drop of blocking buffer (PBS/3% BSA) for 2 hours, the mAb EmG3_IgG1_ was applied at a dilution of 1:10 in blocking buffer for 1 h, followed by a secondary goat-anti-rabbit 10 nm gold conjugate (Aurion, Wageningen, Holland) diluted 1:5 in blocking buffer for 1 h. After several washes in PBS, specimens were fixed in 2% glutaraldehyde phosphate buffer for 10 min, rinsed in water and air-dried. Contrasting by using lead citrate and uranyle acetate was done as previously described (Winzer et al., 2020). Specimens were viewed on a Morgagni Transmission Electron Microscope operating at 80 kV.

## Results

### Species- and stage specificity of monoclonal antibodies

The antigens targeted by the mAbs were evaluated with ELISA by using a panel of 51 native parasitic antigens and 3 recombinant proteins (*E. multilocularis*, *E. granulosus s.l.*, *E. vogeli*, *Taenia* spp., and various helminths). A summary of the antigens and mAb binding is provided in **Table 2**. Our mAbs showed distinct binding patterns in ELISA and potential cross-reactivity only within the genus *Echinococcus*. While mAb Em2G11 is species-specific for *E. multilocularis*, mAb Eg2 is species-specific for *E. granulosus s.l.* All other mAbs labelled antigens of all evaluated *Echinococcus* species including *E. vogeli*. Furthermore, mAb Em18 was reactive with a 65 kDa band in an *E. multilocularis* vesicle fluid western blot. This 65 kDa band corresponds to the full length ‘‘II/3 antigen’’ of *E. multilocularis* (Felleisen and Gottstein, 1993). Moreover, the mAb AgB was reactive with an 8 kDa band in an Antigen B western blot (Poretti et al., 1999) and an *E. multilocularis* vesicle fluid western blot (Müller et al., 2007). A complete table with all antigens and control mAbs, western blots, ELISA internal controls (polyclonal mouse and rabbit sera), and a conjugate control is provided in **Supplementary Table 1**.

### Immunohistochemical stainings on human AE and CE liver sections

**Figure 1:**
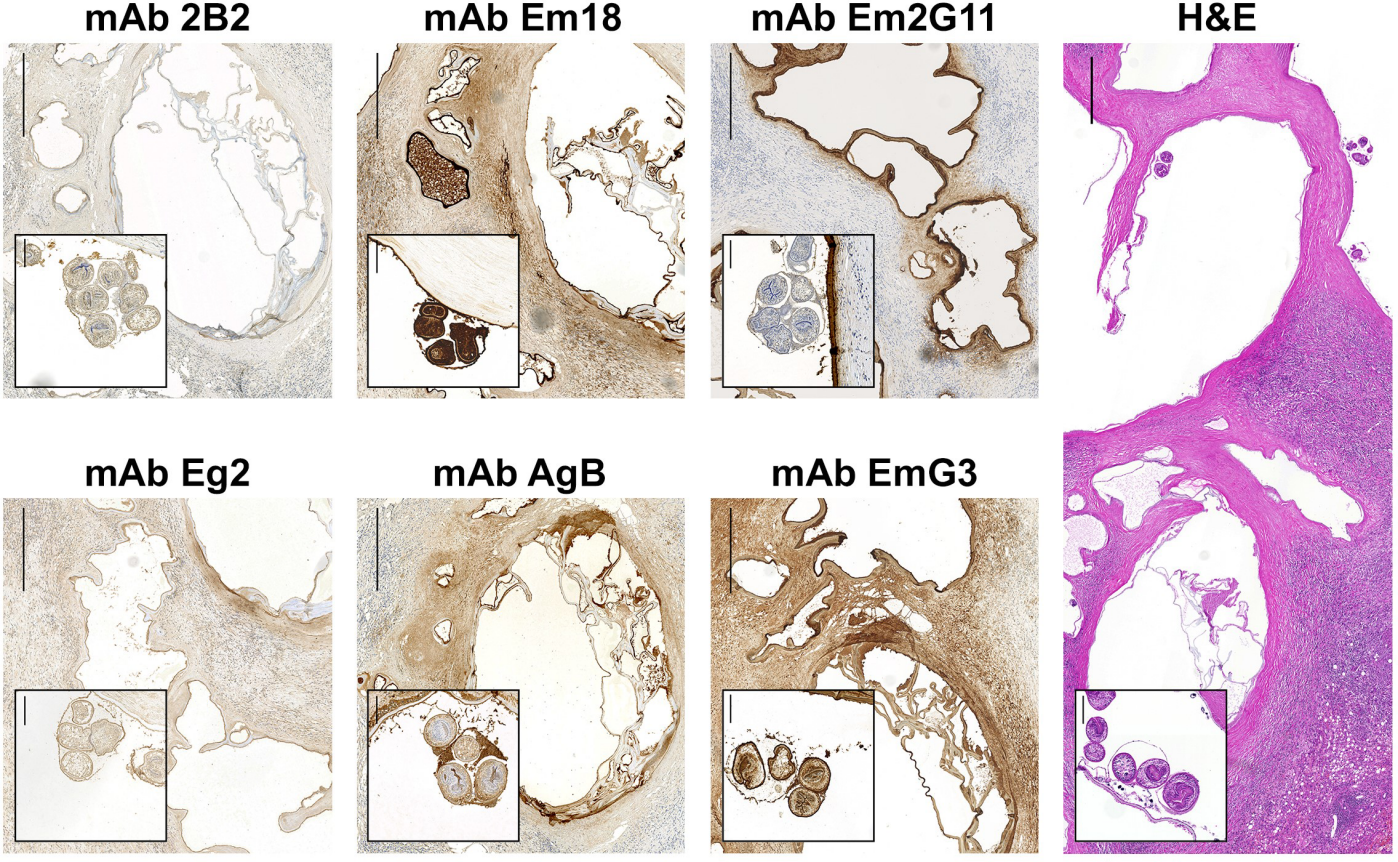
Immunohistochemical stainings (IHC-S) with mAbs on liver sections of a patient with alveolar echinococcosis. H&E, Hematoxylin and eosin staining. Scale bars: 1 mm; insets: 100 μm. The enlarged sections are brood capsules of *E. multilocularis*, surrounded by a germinal layer. A positive staining is connected to a strong brownish color.

**Figure 2:**
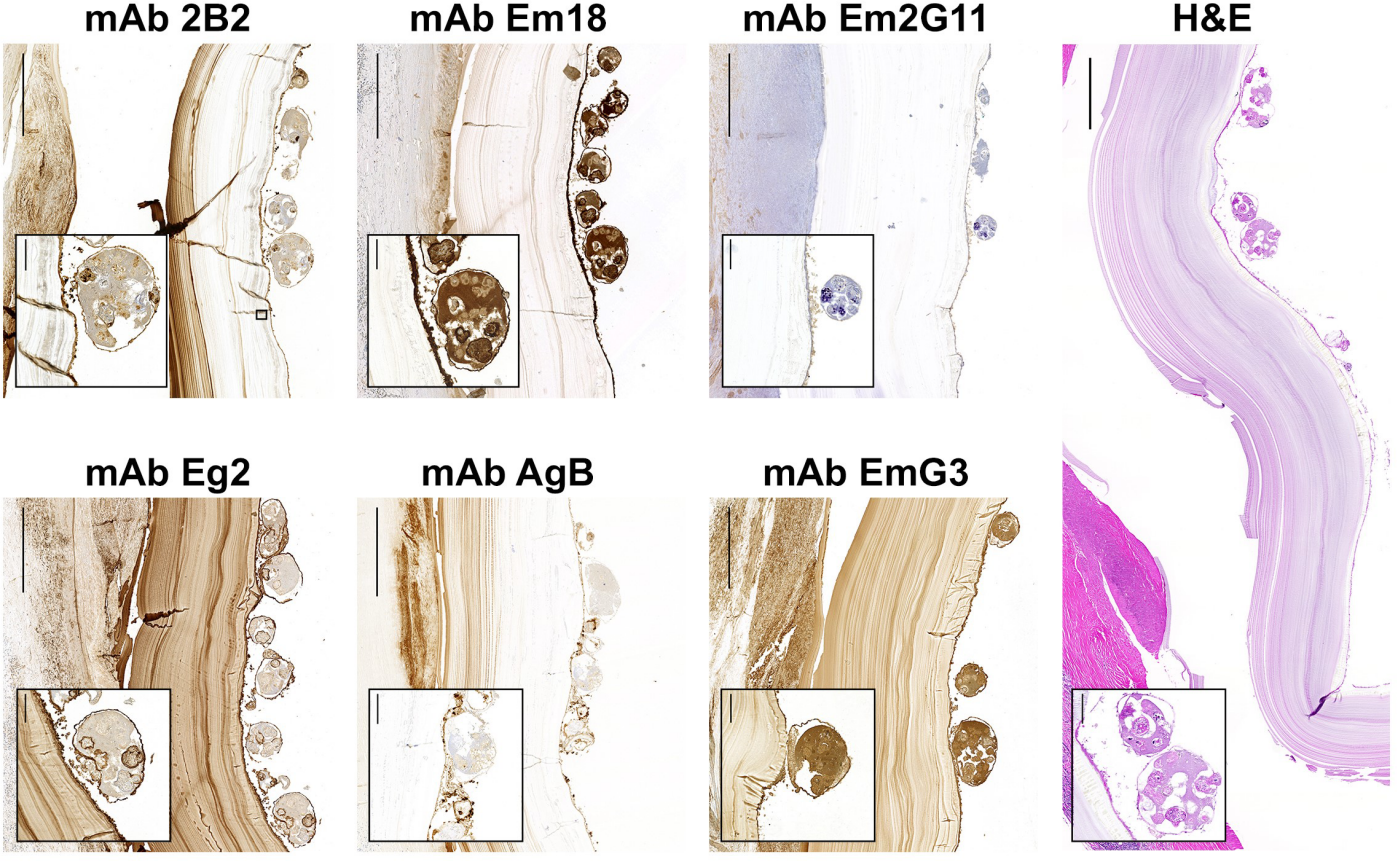
Immunohistochemical stainings (IHC-S) with mAbs on liver sections of a patient with cystic echinococcosis. H&E, haematoxylin and eosin staining. Scale bars: 1 mm; insets: 100 μm. The enlarged sections are brood capsules of *E. granulosus s.l.*, surrounded by a germinal layer. A positive staining is connected to a strong brownish color.

The panel of 7 mAbs and two control mAbs were evaluated in IHC-S (**Table 3**). All mAbs were tested on one AE and one CE liver section from human patients (**Figures 1**, **2**). The results of IHC-S are in consensus with the evaluation of mAbs in ELISA. The mAb Em2G11 and mAb EmG3_IgM_ were already evaluated for their utility in IHC-S by Reinehr et al. (2020). While mAb Em2G11 is species-specific for *E. multilocularis*, mAb EmG3_IgM_ binds to all *Echinococcus* species, including *E. vogeli* (Reinehr et al., 2020). The mAb Em2G11 is predominantly directed against the laminated layer (LL) and spems of *E. multilocularis* and exhibits a weak granular staining pattern in the germinal layer (GL) and in the protoscoleces (**Figures 1** and **2**, mAb Em2G11). The mAb EmG3_IgG1_ showed a wide staining pattern with all parasitic structures of both species, exactly as the already described mAb EmG3_IgM_ (**Figures 1**, **2**, mAb EmG3). The newly produced mAb Em18 strongly labelled cells of the GL, the tegument, rostellum and cellular contents of the protoscoleces of both species (comparable to mAb EmG3_IgG1_), but without staining of the LL nor with spems or spegs (**Figures 1**, **2**, mAb Em18). The mAb AgB and mAb 2B2 were reactive with all parasite structures in both CE and AE including spems and spegs. Both mAb AgB and mAb 2B2 showed a granular staining pattern in the GL, which was most likely correlated to single cells producing these antigens (**Figures 1**, **2**, mAb AgB and mAb 2B2). The mAb 2B2 signal was clearly reduced in the GL of *E. multilocularis*, compared to *E. granulosus s.l*. The mAb Eg2 displayed a species-specific binding pattern for all structural parts of *E. granulosus s.l.*, including spegs. No reaction was observed in liver sections of *E. multilocularis* with mAb Eg2 (**Figures 1**, **2**, mAb Eg2). The control antibodies D.i 36/1 and A.v 2/1/3 exhibited no reactivity in both AE and CE liver sections (**Supplementary Figure 1**).

**Table 3:** Immunohistochemical stainings (IHC-S) with mAbs on liver sections of one alveolar- and one cystic echinococcosis patient.

IHC-S	*E. multilocularis, alveolar echinococcosis*				*E. granulosus s.l., cystic echinococcosis*			
mAb	laminated layer	germinal layer	protoscoleces and brood capsules	spems*	laminated layer	germinal layer	protoscoleces and brood capsules	spegs*
Em2G11	positive	weak granular staining	weak granular staining	positive	negative	negative	negative	negative
EmG3_IgM_	positive	positive	tegument & rostellum, liquid content of brood capsules	positive	positive	positive	tegument & rostellum, liquid content of brood capsules	positive
EmG3_IgG1_	positive	positive	tegument & rostellum, liquid content of brood capsules	positive	positive	positive	tegument & rostellum, liquid content of brood capsules	positive
Em18	negative	positive	tegument & rostellum, cellular content of protoscoleces and brood capsules	negative	negative	positive	tegument & rostellum, cellular content of protoscoleces and brood capsules	negative
AgB	positive	positive	liquid content of brood capsules	positive	positive	positive	liquid content of brood capsules	positive
2B2	positive	weak granular staining	liquid content of brood capsules	positive	positive	positive	liquid content of brood capsules	positive
Eg2	negative	negative	negative	negative	positive	positive	tegument & rostellum, liquid content of brood capsules	positive
D.i 36/1	negative	negative	negative	negative	negative	negative	negative	negative
A.v 2/1/3	negative	negative	negative	negative	negative	negative	negative	negative

*spems and spegs: Small particles of *E. multilocularis* and *E. granulosus*. Spems and spegs are antigenic particles that are stained in the surrounding tissue of the parasite lesions (Reinehr et al., 2020; Ricken et al., 2017) and in bordering lymph nodes (Grimm et al., 2020).

### Binding of mAbs with extravesicular *in vitro* ESP and circulating serum antigens by sandwich ELISA

All mAbs were evaluated for their binding capacity to extravesicular ESP and circulating serum antigens of intermediate and accidental intermediate hosts by sandwich-ELISA (**Tables 4**, **5**). Extravesicular ESP of *E. multilocularis* was recognized by both mAb Em2G11 and mAb EmG3. For extravesicular ESP of *E. granulosus s.s.*, only mAb EmG3 was positive. In gerbil serum with experimental intraperitoneal AE, the mAb Em2G11 and mAb EmG3 were positive in all 8 samples, while mAb AgB was positive in 6 samples and mAb 2B2 in 4 samples. In mice infected experimentally with *E. granulosus s.s.* cysts intraperitoneal, only 2 out of 8 serum samples were positive with mAb EmG3. The detection of circulating antigens for CE in sheep failed and only 1 out of 8 human CE patients were positive for circulating antigens with mAb EmG3. For hosts with naturally acquired AE, both mAb Em2G11 and mAb EmG3 were positive in 2 out of 8 dogs and 2 out of 8 monkeys, while only 1 out of 8 human AE patient had detectable circulating antigen in sandwich-ELISA.

**Table 4:** Antigen binding of mAbs with extravesicular *in vitro* ESPs and circulating serum antigens of *E. multilocularis* metacestode infections by sandwich-ELISA.

E. multilocularis	*in vitro* ESP*	Gerbil**	Dog***	Monkey***	Human***
mAbs	N = 2	N = 8	N = 8	N = 8	N = 8
mAb Em2G11	2	8	2	2	1
mAb EmG3_IgM_	2	8	2	2	1
mAb EmG3_IgG1_	2	8	2	2	1
mAb Em18	0	0	0	0	0
mAb AgB	0	6	0	0	0
mAb 2B2	0	4	0	0	0
mAb Eg2	0	0	0	0	0
mAb D.i 36/1	0	0	0	0	0
mAb A.v 2/1/3	0	0	0	0	0

* Two different isolates (E4 European haplotype (Nakao et al., 2009) and A2 Asian haplotype (Alvarez Rojas et al., 2020)/** Experimental, secondary intraperitoneal infection/*** Natural liver infections/Sandwich ELISA: Coating with mAbs, detection of circulating serum antigens with polyclonal rabbit serum as described in Material & Methods. A cut-off was determined with the OD values of 8 uninfected control samples for each species and ESP of the control medium including 3T3-feeder cells and 10% FBS. The cut-off was set as the mean value of all negatives plus three standard deviations.

**Table 5:** Antigen binding of mAbs with extravesicular *in vitro* ESPs and circulating serum antigens of *E. granulosus s.s.* metacestode infections by sandwich-ELISA.

*E. granulosus s.s.*	*in vitro* ESP*	Mice**	Sheep***	Human***
mAbs	N = 2	N = 8	N = 8	N = 8
mAb Em2G11	0	0	0	0
mAb EmG3_IgM_	2	2	0	1
mAb EmG3_IgG1_	2	2	0	1
mAb Em18	0	0	0	0
mAb AgB	0	0	0	0
mAb 2B2	0	0	0	0
mAb Eg2	0	0	0	0
mAb D.i 36/1	0	0	0	0
mAb A.v 2/1/3	0	0	0	0

* Two different isolates (G1 genotype isolated from sheep cysts)/**Experimental, secondary intraperitoneal infection/*** Natural liver infections/Sandwich ELISA: Coating with mAbs, detection of circulating serum antigens with polyclonal rabbit serum as described in Material & Methods. A cut-off was determined with the OD values of 8 uninfected control samples for each species and ESP of the control medium including 3T3-feeder cells and 10% FBS. The cut-off was set as the mean value of all negatives plus three standard deviations.

### Antigen binding of mAbs with purified *in vitro* EVs and soluble ESP of *Echinococcus* species by sandwich-ELISA

All mAbs were evaluated for their binding capacity to EVs, ESP, and soluble antigens by sandwich-ELISA (**Table 6**). The mAbs EmG3_IgM_ & EmG3_IgG1_ specifically detected purified intravesicular EVs from vesicle fluid of *E. multilocularis* and *E. granulosus s.l.*, and EVs from cyst fluid of *E. vogeli*. None of the tested mAbs were reactive with secreted extravesicular EVs in all species. Soluble extravesicular ESP could be detected by mAb Em2G11, mAb EmG3_IgM_, and mAb EmG3_IgG1_ in *E. multilocularis*. In *E. granulosus s.s.*, soluble extravesicular ESP could be detected by mAb EmG3_IgM_ and mAb EmG3_IgG1_. The mAb Em18 detected crude antigens of all species but not extravesicular ESP nor antigens inside the vesicle or cyst fluid. The mAb AgB and mAb 2B2 were reactive with crude antigens of all *Echinococcus* species and most notably with soluble antigens from the vesicle or cyst fluid. The mAb Eg2 was solely reactive with a vesicle crude antigen of *E. granulosus s.l.*

**Table 6:** Antigen binding of mAbs with purified extravesicular vesicles (EVs) and soluble excretory/secretory products (ESP) of *Echinococcus* species by sandwich-ELISA.

Species/Control	Antigens (Ag)	mAbEm2G11	mAbEmG3_IgM_	mAbEmG3_IgG1_	mAbEm18	mAbAgB	mAb2B2	mAbEg2	mAbA.v 2/1/3	mAbD.i 36/1
*E. multilocularis*	vesicle crude Ag	++++	++++	++++	+	+++	+	–	–	–
*E. multilocularis*	vesicle fluid EVs	–	++++	++++	–	–	–	–	–	–
*E. multilocularis*	extravesicular ESP EVs	–	–	–	–	–	–	–	–	–
*E. multilocularis*	soluble Ag vesicle fluid	–	++++	++++	–	++++	++++	–	–	–
*E. multilocularis*	soluble Ag extravesicular ESP	++++	++++	++++	–	–	–	–	–	–
*E. granulosus s.s.*	vesicle crude Ag	–	++++	++++	+	++++	+	++++	–	–
*E. granulosus s.s.*	vesicle fluid EVs	–	++++	++++	–	–	–	–	–	–
*E. granulosus s.s.*	extravesicular ESP EVs	–	–	–	–	–	–	–	–	–
*E. granulosus s.s.*	soluble Ag vesicle fluid	–	++++	++++	–	++++	+++	–	–	–
*E. granulosus s.s.*	soluble Ag extravesicular ESP	–	+++	+++	–	–	–	–	–	–
*E. vogeli*	cyst crude Ag	–	++++	++++	+	++++	+	–	–	–
*E. vogeli*	cyst fluid EVs	–	++++	++++	–	–	–	–	–	–
*E. vogeli*	soluble Ag cyst fluid	–	++++	++++	–	++	–	–	–	–
control medium*	extracellular ESP EVs	–	–	–	–	–	–	–	–	–
control medium*	soluble Ag extracellular ESP	–	–	–	–	–	–	–	–	–

ELISA OD: - (0.0-0.05)/+ (0.05-0.25)/++ (0.25-0.5)/+++ (0.5-1.0)/++++ (>1.0)/Sandwich-ELISA: Coating with mAbs, detection of antigen fractions with polyclonal rabbit serum as described in Material & Methods./*Control medium from 3T3-feeder cells including 10% fetal bovine serum.

### Immunogold-labelling of EVs of *in vitro* cultured *E. multilocularis* metacestodes

**Figure 3:**
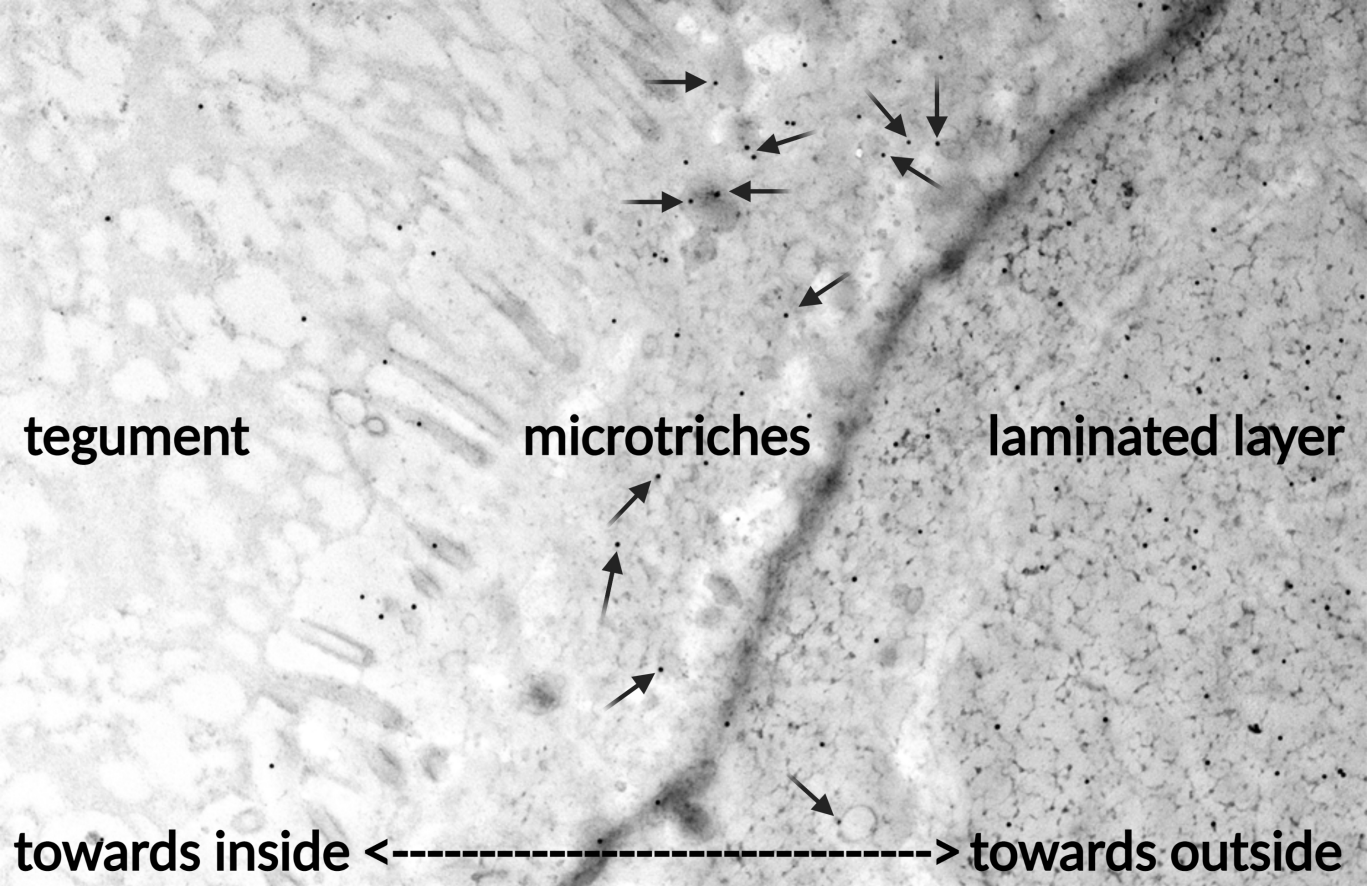
Transmission electron microscopy (TEM) of mAb EmG3_IgG1_ on extracellular vesicles (EVs) of *E. multilocularis in vitro* vesicles. Arrows: Nano-gold labelling of the mAb EmG3_IgG1_ confirms the binding to exosomes or EVs in the border of the laminated layer to the tegument and towards the inside of the vesicles and vesicle fluid.

Immunogold-labelling by employing the mAb EmG3_IgG1_ was performed on ultrathin sections of *in vitro* cultured metacestodes of *E. multilocularis*. The mAb EmG3_IgG1_ specifically decorated EVs that emerged from the microtriches and were released from the tegumental border into the matrix of the laminated layer. The arrows in **Figure 3** point towards the nano-gold conjugates binding to the mAb EmG3_IgG1_. Overall, the labelling is more intense in the region of the laminated layer where EVs are clearly visible, such as closer to the tegument, and staining intensity is diminished further away from the tegument, and basically absent at the outer border of the laminated layer (data not shown).

## Discussion

Over the recent decades, research on the biological characterization of several *Echinococcus* species have advanced to sophisticated secretome analyses, transcriptomics, and whole genome sequencing (Brehm and Koziol, 2017). Nevertheless, basic biological functions and parasite-host interactions of the larval stage (metacestode) of *Echinococcus* species are to date still limited due to the lack of markers for enhanced structural visualizations. In this respect, we have evaluated a panel of 7 monoclonal antibodies (mAbs) directed against the major known diagnostic antigens of *Echinococcus* species and determined the localization of these antigens with respect to their putative function in host-parasite interactions.

We have produced a new mAb Em18, which is reactive with both recombinant Em18 and II/3-10 antigens. We are confident that this mAb is directed against the shared diagnostic epitope of both antigens. It is of note, that the recombinant II/3-10 and Em18 antigens were so far considered to be specific for *E. multilocularis*. Up to date, one study has indicated the strong homology in the II/3-10 gene of *E. multilocularis* and *E. granulosus s.l.* of 98.45% (Felleisen and Gottstein, 1994). Nonetheless this striking homology, the different antibody responses of AE and CE patients against the Em18 or II/3-10 antigen became evident in several studies. In Swiss AE patients, the Em18 antigen has a sensitivity of 82-92%, while in CE patients, only 6-13% have antibodies against the Em18 antigen (Gottstein et al., 1993; Kronenberg et al., 2022; Schweiger et al., 2012). We could confirm the localization of the Em18 antigen in cells of the germinal layer (GL) and protoscoleces in both AE (**Figure 1**) and CE patients (**Figure 2**) by IHC-S. In our opinion, the lower sero-positivity in CE patients could be suppressed by the wider laminated layer (LL) and the non-invasive growth of *E. granulosus s.l.* metacestodes, compared to the infiltrative growth of *E. multilocularis* with a narrower protective LL. While not yet confirmed, it seems plausible that CE patients could become sero-positive in Em18 serology after cyst rupture. A previous study (Kronenberg et al., 2022) has shown that 8 out of 64 confirmed CE patients (12.5%) were positive in the Em18 ELISA. However, none of these 8 CE patients had a report of cyst rupture or dissemination and all ELISA ODs were barely over the cut-off, which on itself was very low (OD cut-off: 0.06; mean value of all CE patients, OD: 0.1 (0.06-0.16)). Currently, the Em18 antigen has been mainly used for the follow-up of AE patients under long-term benzimidazole therapy. Negativity of anti-Em18 antibodies for at least two years is one of the parameters used to determine, if benzimidazole therapy in inoperable AE patients can be stopped (Ammann et al., 2015; Deibel et al., 2022). Thereby, the Em18 antigen seems to be vital for biological functions and the viability of especially *E. multilocularis* metacestodes. As our mAb Em18 is binding to all cells of the GL, it would likely be expected to bind to primary- or stem cells as well. However, further studies are required to confirm this. Another study has performed IHC-S with a mAb against Em10 in a human *E. vogeli* infection (Stijnis et al., 2015). The mAb against Em10 showed a positive staining in the GL and protoscoleces of *E. multilocularis* and *E. granulosus s.l.*, but only a faint staining pattern in protoscoleces of *E. vogeli*. It remains unknown, if our mAb Em18 is directed against the same epitope.

Moreover, our study has produced a new monoclonal antibody (mAb Eg2), which is species-specific for *E. granulosus s.l.* A species-specific epitope was so far exclusively known from *E. multilocularis*, where the Em2 antigen can be used for a specific diagnosis of AE patients and the corresponding mAb Em2G11 in species-specific immunohistochemical stainings (Deplazes and Gottstein, 1991; Kronenberg et al., 2022; Reinehr et al., 2020). We are anticipating similar results with mAb Eg2 in ongoing studies for species-specific IHC-S for *E. granulosus s.l.* metacestodes. In addition, we have evaluated the affinity purified Eg2 antigen as a species-specific serological marker in 64 confirmed CE patients in a preliminary study (Kronenberg et al., 2022). However, in these unpublished observations, the Eg2 antigen was not superior by comparing sensitivity and specificity to widely used cyst fluid or protoscolex antigens of *E. granulosus s.l.*

In our study, we also aimed at understanding the secretion of ESP in *Echinococcus* species *in vitro* and subsequently in the blood of intermediate hosts (**Tables 4** and **5**). Therefore, we have each used two different *in vitro* isolates of *E. multilocularis* and *E. granulosus s.s.*, respectively. Our results indicate, that the antigens Em2 and EmG3 are secreted towards the extravesicular proximity of cultured *in vitro* vesicles as soluble antigens (**Table 6**). In addition, these two antigens and corresponding mAbs were the only candidates with a potential to detect circulating antigens in blood of intermediate hosts with AE (humans, dogs, monkeys) and CE (humans, mice). However, the sensitivity for the detection of circulating antigens by ELISA in all species was very low (max. 25% for animals and 13% for humans). This could be caused by a too low concentration of circulating antigens, by immune-complexes or by using an unsuitable detection method (conventional sandwich-ELISA). Similar results were already described in other studies, where the sensitivity for the detection of circulating antigens in blood of CE patients ranged only between 3 to 33% in sandwich-ELISA by using polyclonal antibodies (Craig, 1986; Craig and Nelson, 1984; Gottstein, 1984). In our opinion, future research should focus on improving the limit of detection (e.g. biosensors) to identify circulating antigens in blood with the mAb Em2G11 and mAb EmG3.

In a previous study, the Em2 antigen could be detected by sandwich-ELISA in the supernatant of *in vitro* cultured vesicles, in vesicle crude antigen, and in the vesicle fluid (Hemphill and Gottstein, 1995). Our study could confirm these results for the most part, but the vesicle fluid was negative for the Em2 antigen in sandwich-ELISA in two different isolates. In our opinion, we expect the Em2 antigen only to be secreted towards the outside of vesicles and as ESP, but the related EmG3 antigen is secreted both towards the inside and outside of cultured vesicles. Another interesting observation in mice infected with *E. multilocularis* is, that the Em2 antigen represents a T-cell independent epitope (Gottstein et al., 2006). This could be correlated to an ineffective immune attack against the parasite in mice, but most likely also in humans, by secreting soluble Em2 antigen into the environment as observed in our cultured *in vitro* vesicles.

Moreover, the comparison of IHC-S (spems & spegs) (**Table 3**) and the detection of ESP by sandwich-ELISA, revealed some conflicting results (**Table 6**). In the literature, spems and spegs are described as small particles of both *E. multilocularis* and *E. granulosus s.l.* These particles are considered as antigenic remnants, that are found outside of the LL in the tissue surrounding the lesions (Reinehr et al., 2020; Ricken et al., 2017) and in the bordering lymph nodes (Grimm et al., 2020). Their role as being a part of the secreted ESP is so far unknown. In IHC-S, all mAbs except the mAb Em18 are binding spems or spegs, while for extravesicular ESP fractions, only mAb Em2G11 and mAb EmG3 are positive in sandwich-ELISA. Therefore, spems and spegs seem unsuitable characteristics to observe which antigens are secreted and which antigens are depleted or carried away by immune cells.

Furthermore, the mAb AgB and mAb 2B2 reacted exclusively with soluble vesicle fluid, and the corresponding antigens seem not to be secreted as extravesicular ESP towards the outside of vesicles. Hereby we could also confirm that the *in vitro* cultured vesicles did not leak vesicle fluid and could have masked the ESP fractions with Antigen B. In this case, both mAb AgB and mAb 2B2 would have been positive with extravesicular ESP fractions in sandwich-ELISA. Furthermore, we could illustrate, that both antigen B and antigen 2B2 seem to be produced by single cells in the germinal layer of *Echinococcus* spp., which was so far vaguely described (Siles-Lucas et al., 2017a).

In our study, we purified extracellular vesicles (EVs) from cultured vesicles of *E. multilocularis* and *E. granulosus s.s.*, and from cyst fluid of *E. vogeli* metacestodes. Our results indicate, that EVs are both secreted towards the outside of vesicles (extravesicular ESP) and towards the inside of the vesicles (intravesicular vesicle fluid). Both concentration and particle sizes of extravesicular and intravesicular EV fractions were similar for all evaluated *Echinococcus* species (**Supplementary Table 2**). Interestingly, only the mAb EmG3_IgM_ and mAb EmG3_IgG1_ were directed against intravesicular EV fractions of all *Echinococcus* species but not reactive with extravesicular EV fractions. It remains unknown if the mAb binding is directed against EVs or EV associated proteins (EV-corona proteins). By transmission electron microscopy, the mAb binding to EVs could be confirmed on the ultrastructural level (**Figure 3**). This observation points out, that EVs could be different inside the vesicles (intravesicular fluid), compared to secreted EVs in the extravesicular ESP. This has been already described in another study (Ancarola et al., 2020). As we have not detected EVs in the extravesicular ESP with our mAb panel, new mAbs should be generated against secreted EV fractions, to have a potential to detect circulating EVs in the blood of intermediate hosts. Nevertheless, the mAb EmG3_IgM_ and mAb EmG3_IgG1_ should be evaluated as EV markers for intravesicular EVs in continuous studies for all *Echinococcus* species.

Concerning the aim of evaluating a panel of mAbs for the characterization of various biological functions in *Echinococcus* species, it is relevant to also acknowledge the limitations of the current study. Mostly single or double replicates were used in our analysis and not triplicates. Nonetheless, despite using single or double replicates, we could demonstrate that our results were in agreement between all methods. Direct ELISA could be confirmed by sandwich ELISA, and the results in IHC-S were comparable to ELISA. Moreover, it remains to be studied whether the ESP fractions of *Echinococcus* spp. could differ between *in vitro* and *in vivo*. Whereas the nature and extent of the possible influence of host-parasite and parasite-host interactions on the secretion of ESP *in vivo* remains to be further explored as well.

## Conclusions

To conclude, our mAbs represent valuable inaugurating research tools for the diagnosis and biological characterization of the most important *Echinococcus* species. Combined with the already established mAb Em2G11 and mAb EmG3, the new mAbs will be evaluated in further studies for an improved species diagnosis (mAb Eg2) or as viability markers by immunohistochemistry including binding to primary- or stem cells (mAb Em18). Furthermore, the mAbs produced in the current study could serve as potential new EV markers (mAb EmG3) or be used in fluorescence-activated cell sorting (FACS) for cell-based assays and drug screenings. Further applications of our mAb panel potentially lies in serology (biosensor), by detecting circulating parasite ESP with a lower limit of detection. Moreover, our mAbs could provide more insights into parasite-host interactions and pathogenesis of echinococcosis, especially when used in combination with different cellular and cytokine immunological markers. All mAbs are available for further research applications on request by Peter Deplazes at the Institute of Parasitology in Zurich, Switzerland.

## Data availability statement

The original contributions presented in the study are included in the article/**Supplementary Material**. Further inquiries can be directed to the corresponding authors.

## Ethics statement

The studies involving human participants were reviewed and approved by the internal review board of the University Hospital Zurich and the Cantonal Ethics Committee of Zurich, Switzerland (KEK-ZH-Nr. 2015-0498), and KEK-ZH-Nr. 2020-0495. The patients/participants provided their written informed consent to participate in this study. The animal study was reviewed and approved by the cantonal veterinary office in Zurich, Switzerland (approval No: ZH235/19, ZH068/19, and ZH111/18).

## Author contributions

Conceptualization: PK and PD. Methodology: PK, MR, RE, AH, and PD. Data curation: PK, MR, RE, AH, and PD. Investigation: PK, MR, RE, SH, TL, AH, and PD. Resources: PK, MR, RE, SH, TL, AW, AD, BM, BG, NM, AH, and PD. Funding acquisition: PD. Writing—original draft: PK and PD. Writing— review and editing: PK, MR, RE, SH, TL, AW, AD, BM, BG, NM, AH, and PD. All authors contributed to the article and approved the submitted version.
