# Supplementary material for: Monoclonal antibody-based localization of major diagnostic antigens in metacestode tissue, excretory/secretory products, and extracellular vesicles of Echinococcus species
Source: Front Cell Infect Microbiol. 2023 Mar 16;13:1162530. doi: 10.3389/fcimb.2023.1162530 (PMC10061086; doi:10.3389/fcimb.2023.1162530)
Supplement: Supplementary file 1 [file DataSheet_1.docx]

Supplementary Material

**Monoclonal antibody-based localization of major diagnostic antigens in metacestode tissue, excretory/secretory products, and extracellular vesicles of *Echinococcus* species**

**Philipp A. Kronenberg^1/2*^, Michael Reinehr^3^, Ramon Marc Eichenberger^1^, Sina Hasler^1^, Teivi Laurimäe^1^, Achim Weber^3^, Ansgar Deibel^4^, Beat Müllhaupt^4^, Bruno Gottstein^5^, Norbert Müller^5/6^, Andrew Hemphill^6^, Peter Deplazes^1/4*^**

*** Correspondence:**Philipp Kronenberg, Peter Deplazes
[philipp.kronenberg@gmail.com](mailto:philipp.kronenberg@gmail.com), peter.deplazes@uzh.ch

| 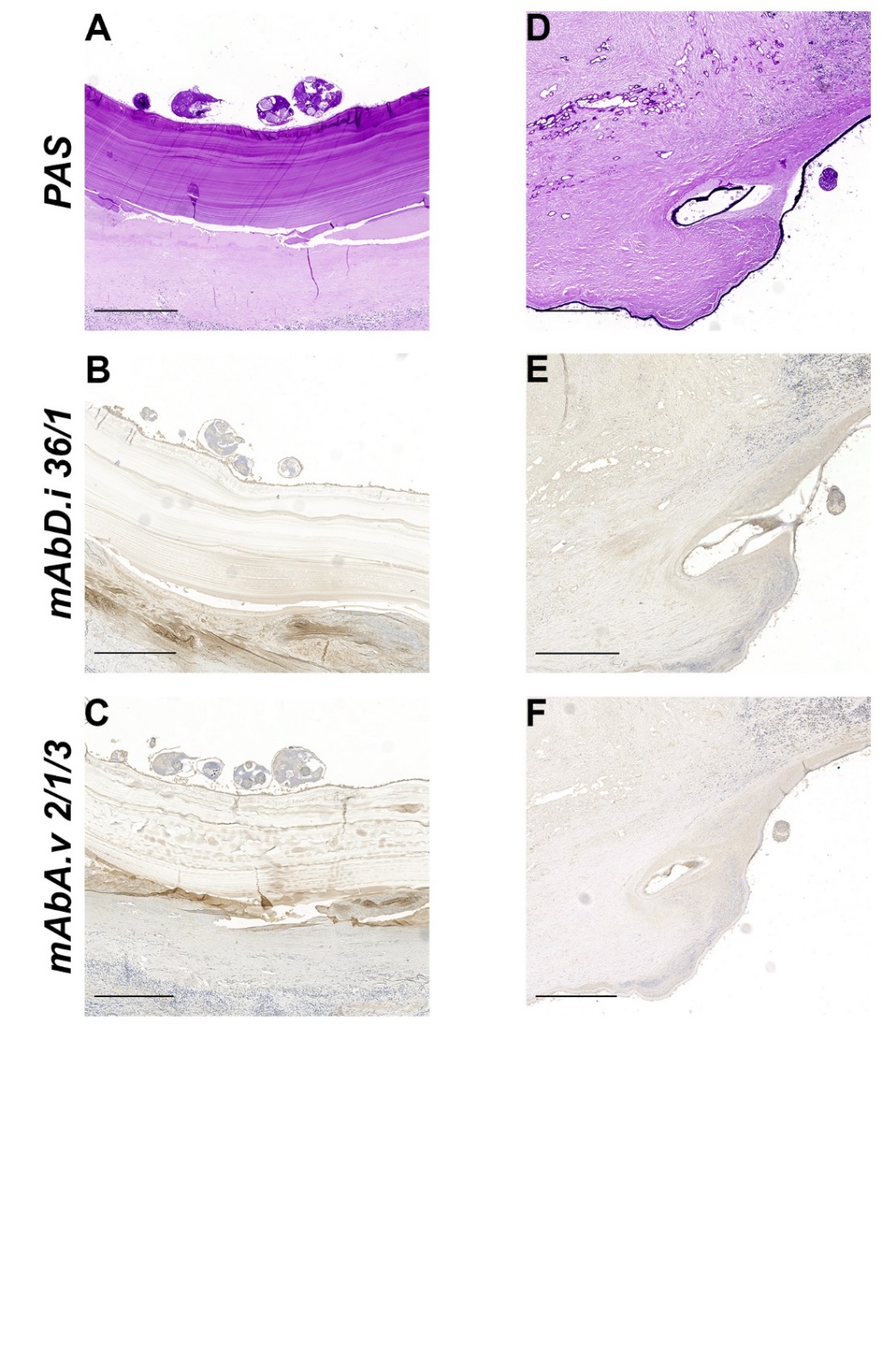**Supplementary figure 1.** Control mAbs: Immunohistochemical- and PAS staining on human alveolar- and cystic echinococcosis liver sections  PAS: Periodic acid–Schiff staining. Scale bars: 1 mm. Both control mAb D.i 36/1 (IgG1) and mAb A.v 2/1/3 (IgM) were tested in IHC-S on a human liver section with *E. granulosus s.l.* (A-C) and *E. multilocularis* (D-F).  **Supplementary table 1.** Binding of mAbs and sera on 54 parasitic antigens | | | | | | | | | | | | | |
| --- | --- | --- | --- | --- | --- | --- | --- | --- | --- | --- | --- | --- | --- |
|  |  | **conjugate control** | **serum mouse AE ^§^** | **polyclonal rabbit serum^#^** | **mAb Em2G11** | **mAb EmG3** | **mAb EmG3** | **mAb Em18** | **mAb AgB** | **mAb 2B2** | **mAb Eg2** | **mAb D.i 36/1** | **mAb A.v 3/1/2** |
| **Species, G = genotype** | **Origin and stage of antigens (Ag)** |  |  |  | **IgG1** | **IgM** | **IgG1** | **IgG1** | **IgG1** | **IgG1** | **IgM** | **IgG1** | **IgM** |
| *E. multilocularis* | human, metacestode crude Ag | - | +++ | ++++ | +++ | +++ | +++ | + | ++ | + | - | - | - |
| *E. multilocularis* | monkey, metacestode crude Ag | - | +++ | ++++ | ++++ | ++++ | ++++ | + | + | - | - | - | - |
| *E. multilocularis* | dog, metacestode crude Ag | - | + | +++ | + | + | + | + | ++++ | + | - | - | - |
| *E. multilocularis* | rat, metacestode crude Ag | - | ++ | ++++ | ++++ | ++++ | +++ | + | + | - | - | - | - |
| *E. multilocularis* | *in vitro*, vesicle somatic Ag* | - | ++++ | ++++ | +++ | ++++ | +++ | ++ | ++ | + | - | - | - |
| *E. multilocularis* | *in vitro*, vesicle somatic Ag** | - | +++ | ++++ | +++ | +++ | +++ | ++ | +++ | + | - | - | - |
| *E. multilocularis* | *in vitro*, pure vesicle fluid* | - | ++++ | ++++ | - | + | + | - | ++++ | ++ | - | - | - |
| *E. multilocularis* | *in vitro*, pure vesicle fluid** | - | +++ | +++ | - | + | + | - | +++ | + | - | - | - |
| *E. multilocularis* | affinity purified Em2G11* | - | ++++ | ++++ | ++++ | ++++ | +++ | - | - | - | - | - | - |
| *E. multilocularis* | gerbil, protoscolex integument Ag*** | - | ++++ | ++++ | - | +++ | +++ | ++ | - | - | - | - | - |
| *E. multilocularis* | gerbil, protoscolex crude Ag | - | ++ | +++ | + | +++ | ++ | + | - | - | - | - | - |
| *E. multilocularis* | *in vitro*, vesicles ESP Ag | - | +++ | +++ | ++ | ++++ | ++++ | - | - | - | - | - | - |
| *E. multilocularis* | fox, adult crude Ag | - | ++ | ++++ | - | - | - | + | - | - | - | - | - |
| *E. multilocularis* | fox, adult ESP Ag | - | +++ | +++ | - | - | - | + | - | - | - | - | - |
| *E. multilocularis* | fox, oncosphere ESP Ag | - | + | +++ | + | + | + | + | - | - | - | - | - |
| *E. multilocularis* | recombinant, Em18 | - | - | - | - | - | - | ++++ | - | - | - | - | - |
| *E. multilocularis* | recombinant, EmII/3-10 | - | - | - | - | - | - | ++++ | - | - | - | - | - |
| *E. multilocularis* | *in vitro*, vesicle fluid, western blot**** | - | N/A | N/A | - | - | - | 65 kDa | 8 kDa | - | - | - | - |
| *E. granulosus,* G1-3 | human, metacestode crude antigen | - | ++++ | ++++ | - | ++++ | ++++ | + | + | + | ++ | - | - |
| *E. granulosus,* G1-3 | sheep, metacestode crude antigen | - | ++++ | ++++ | - | ++++ | ++++ | - | +++ | + | ++ | - | - |
| *E. intermedius,* G7 | pig, metacestode crude antigen | - | +++ | ++++ | - | ++++ | ++++ | - | + | + | ++ | - | - |
| *E. granulosus,* G1-3 | *in vitro*, vesicle somatic Ag | - | ++++ | ++++ | - | ++++ | ++++ | + | + | - | +++ | - | - |
| *E. granulosus,* G1-3 | *in vitro*, pure vesicle fluid | - | +++ | +++ | - | + | +++ | - | +++ | + | - | - | - |
| *E. granulosus,* G1-3 | *in vitro*, vesicles ESP Ag | - | + | + | - | ++ | ++ | - | - | - | - | - | - |
| *E. granulosus,* G1-3 | sheep, protoscolex crude Ag | - | ++++ | ++++ | - | +++ | +++ | ++ | + | - | + | - | - |
| *E. granulosus,* G1-3 | sheep, cyst fluid | - | ++++ | ++++ | - | +++ | ++++ | - | ++++ | ++ | - | - | - |
| *E. equinus,* G4 | horse, cyst fluid | - | +++ | +++ | - | + | + | - | + | + | - | - | - |
| *E. ortleppi,* G5 | cattle, cyst fluid | - | ++++ | ++++ | - | + | +++ | - | +++ | + | - | - | - |
| *E. intermedius,* G6 | camel, cyst fluid | - | ++++ | ++++ | - | +++ | +++ | - | ++++ | +++ | - | - | - |
| *E. intermedius,* G7 | pig, cyst fluid | - | ++++ | ++++ | - | ++++ | ++++ | - | ++++ | + | - | - | - |
| *E. granulosus,* G1-3 | sheep, purified Antigen B | - | ++ | ++++ | - | +++ | +++ | - | +++ | ++++ | - | - | - |
| *E. granulosus,* G1-3 | dog, adult integument Ag | - | + | +++ | - | - | - | + | - | - | - | - | - |
| *E. granulosus* | recombinant, 2B2 | - | - | - | - | - | - | - | - | ++++ | - | - | - |
| *E. granulosus,* G1-3 | sheep, cyst fluid, western blot***** | - | N/A | N/A | - | - | - | - | 8 kDa | - | - | - | - |
| *E. vogeli* | gerbil, metacestode crude antigen | - | ++ | ++ | - | ++ | +++ | + | + | + | - | - | - |
| *Taenia hydatigena* | sheep, metacestode crude Ag | - | + | +++ | - | - | - | - | - | - | - | - | - |
| *Taenia hydatigena* | sheep, metacestode ESP Ag | - | + | ++ | - | - | - | - | - | - | - | - | - |
| *Taenia solium* | pig, metacestode crude Ag | - | ++ | +++ | - | - | - | - | - | - | - | - | - |
| *Taenia saginata* | cattle, metacestode crude Ag | - | + | +++ | - | - | - | - | - | - | - | - | - |
| *Taenia saginata* | cattle, metacestode ESP Ag | - | + | ++ | - | - | - | - | - | - | - | - | - |
| *Taenia multiceps* | sheep, metacestode crude Ag | - | ++ | +++ | - | - | - | - | - | - | - | - | - |
| *Taenia crassiceps* | mouse, metacestode crude Ag | - | +++ | +++ | - | - | - | - | - | - | - | - | - |
| *Fasciola hepatica* | cattle, adult worm crude Ag | - | +++ | + | - | - | - | - | - | - | - | - | - |
| *Dicrocoelium dendriticum* | sheep, adult worm crude Ag | - | +++ | +++ | - | - | - | - | - | - | - | - | - |
| *Shistosoma mansoni* | human, adult worm crude Ag | - | + | +++ | - | - | - | - | - | - | - | - | - |
| *Litomosoides carinii* | cotton rat, adult worm crude Ag | - | ++++ | +++ | - | - | - | - | - | - | - | - | + |
| *Onchocerca jakutensis* | deer, adult worm crude Ag | - | ++ | +++ | - | - | - | - | - | - | - | - | + |
| *Trichinella spiralis* | mouse, larval crude Ag | - | ++++ | + | - | - | - | - | - | - | - | - | - |
| *Strongyloides ratti* | rat, larval crude Ag | - | + | + | - | - | - | - | - | - | - | - | + |
| *Toxocara canis* | fox, eggs, larval ESP Ag | - | ++++ | - | - | - | - | - | - | - | - | - | - |
| *Dirofilaria immitis* | dog, adult worm crude Ag | - | ++++ | + | - | - | - | - | - | - | - | ++++ | - |
| *Dirofilaria repens* | dog, adult worm crude Ag | - | +++ | + | - | - | - | - | - | - | - | ++ | - |
| *Ascaris lumbricoides* | human, adult worm crude Ag | - | + | + | - | - | - | - | - | - | - | - | - |
| *Angiostrongylus vasorum* | fox, adult worm crude Ag | - | - | + | - | - | - | - | - | - | - | - | +++ |

ELISA OD: - (0.0-0.05) / + (0.05-0.25) / ++ (0.25-0.5) / +++ (0.5-1.0) / ++++ (>1.0) / N/A: Not available / * European haplotype, E4 (Nakao et al., 2009) / ** Kyrgyz haplotype, A2 (Alvarez Rojas et al., 2020) / *** Em2G11 depleted (Kronenberg et al., 2022) / **** Western blot (Müller et al., 2007) / ***** Western blot (Poretti et al., 1999) / § Serum of mouse infected with 500 *E. multilocularis* eggs and progressive alveolar echinococcosis (AE) / # Polyclonal rabbit serum, immunization with *E. multilocularis* and *E. granulosus s.s.* vesicle crude antigen (as used for Sandwich-ELISA)

**Supplementary table 2.** Extracellular vesicles (EVs) of *Echinococcus* species

| **Parasite/Control** | **EV origin** | **EV fractions** | **EV median size (nm)** | **EV concentration (particles/mL)** | **EV BCA (µg/ml)** |
| --- | --- | --- | --- | --- | --- |
| *E. multilocularis* | *in vitro* vesicle fluid | 3 | 80.5 +/- 41.1 | 1.00E+10 | 12.70 |
| *E. multilocularis* | *in vitro* vesicle fluid | 4 | 98.0 +/- 57.0 | 1.20E+10 | 14.22 |
| *E. multilocularis* | *in vitro* vesicle fluid | 5 | 89.1 +/- 67.2 | 1.10E+10 | 14.59 |
| *E. multilocularis* | *in vitro* vesicle fluid | 6 | 105.8 +/-73.1 | 9.10E+10 | 20.47 |
| *E. multilocularis* | *in vitro* vesicle fluid | 7* | 104.0 +/- 55.0 | 9.80E+10 | 27.8 |
| *E. multilocularis* | *in vitro* vesicle fluid | 8* | 95.5 +/- 58.6 | 1.40E+11 | 37.64 |
| *E. multilocularis* | *in vitro* ESP | 3 | 111.2 +/- 91.3 | 3.40E+10 | 45.35 |
| *E. multilocularis* | *in vitro* ESP | 4 | 122.5 +/-65.4 | 2.10E+10 | 19.03 |
| *E. multilocularis* | *in vitro* ESP | 5 | 110 +/- 85.4 | 2.20E+10 | 22.25 |
| *E. multilocularis* | *in vitro* ESP | 6 | 130 +/-78.4 | 2.80E+10 | 23.66 |
| *E. multilocularis* | *in vitro* ESP | 7* | 123.6 +/- 56.3 | 5.90E+10 | 27.81 |
| *E. multilocularis* | *in vitro* ESP | 8* | 94.0 +/-55.8 | 2.00E+11 | 36.05 |
| *E. granulosus s.s.* | *in vitro* vesicle fluid | 3 | 91.5 +/- 62.3 | 5.40E+10 | 20.11 |
| *E. granulosus s.s.* | *in vitro* vesicle fluid | 4 | 98.8 +/- 75.9 | 6.60E+10 | 20.11 |
| *E. granulosus s.s.* | *in vitro* vesicle fluid | 5 | 99.6 +/- 82.7 | 1.10E+11 | 19.03 |
| *E. granulosus s.s.* | *in vitro* vesicle fluid | 6 | 132.7 +/- 66.4 | 6.40E+10 | 22.61 |
| *E. granulosus s.s.* | *in vitro* vesicle fluid | 7* | 116.6 +/- 62.9 | 1.10E+11 | 30.49 |
| *E. granulosus s.s.* | *in vitro* vesicle fluid | 8* | 104.4 +/- 77.1 | 1.90E+11 | 39.21 |
| *E. granulosus s.s.* | *in vitro* ESP | 3 | 116.9 +/- 89.9 | 2.80E+10 | 32.81 |
| *E. granulosus s.s.* | *in vitro* ESP | 4 | 131.2 +/- 77.0 | 3.30E+10 | 31.49 |
| *E. granulosus s.s.* | *in vitro* ESP | 5 | 101.4 +/- 75.7 | 5.00E+10 | 28.18 |
| *E. granulosus s.s.* | *in vitro* ESP | 6 | 99.3 +/- 60.1 | 7.50E+10 | 31.49 |
| *E. granulosus s.s.* | *in vitro* ESP | 7* | 105.2 +/- 86.0 | 5.40E+10 | 34.76 |
| *E. granulosus s.s.* | *in vitro* ESP | 8* | 91.4 +/- 65.3 | 2.50E+11 | 46.85 |
| *E. vogeli* | cyst fluid from gerbils | 3 | N/A | N/A | 32.34 |
| *E. vogeli* | cyst fluid from gerbils | 4 | N/A | N/A | 42.21 |
| *E. vogeli* | cyst fluid from gerbils | 5 | N/A | N/A | 40.97 |
| *E. vogeli* | cyst fluid from gerbils | 6 | N/A | N/A | 52.04 |
| *E. vogeli* | cyst fluid from gerbils | 7* | N/A | N/A | 56.95 |
| *E. vogeli* | cyst fluid from gerbils | 8* | N/A | N/A | 100.88 |
| 3T3-feeder cells | culture medium/10% FBS | 3 | 102.3 +/- 56.3 | 1.40E+11 | 195.25 |
| 3T3-feeder cells | culture medium/10% FBS | 4 | 106.1 +/- 57.2 | 2.10E+11 | 126.14 |
| 3T3-feeder cells | culture medium/10% FBS | 5 | 104.6 +/- 57.7 | 5.50E+11 | 72.71 |
| 3T3-feeder cells | culture medium/10% FBS | 6 | 100.5 +/- 59.3 | 3.50E+12 | 128.29 |
| 3T3-feeder cells | culture medium/10% FBS | 7* | 104.7 +/- 65.6 | 1.60E+12 | 130.05 |
| 3T3-feeder cells | culture medium/10% FBS | 8* | 107.7 +/- 63.2 | 2.30E+12 | 117.28 |

* Fractions 7 and 8 were pooled and used in Sandwich ELISA with mAbs as shown in table. 6
